# Nonoperative Management of Proximal Tibiofibular Joint Synostosis after Tibial Intramedullary Nailing

**DOI:** 10.1155/2019/2423010

**Published:** 2019-05-30

**Authors:** David C. Ebbott, Alexander J. Johnson, Christopher Haydel

**Affiliations:** Temple University, Department of Orthopaedics and Sports Medicine, 3401, N. Broad St. Zone B, 6th Floor, Philadelphia, PA 19140, USA

## Abstract

We report the case of a 28-year-old male semiprofessional basketball player who presented to an outside hospital with nonhealing stress fractures for which he underwent tibial intramedullary nailing (IMN). Two weeks after surgery, he developed pain proximal and lateral to the knee. As he returned to play, the pain worsened with jumping and lateral movement and improved with rest. He presented to our hospital one year after the operation with the same unresolved pain. Imaging one year after the surgery revealed proximal tibiofibular joint (TFJ) synostosis aligned with the drill path. Literature review showed that rare noncongenital cases of proximal TFJ synostosis cases were most often treated nonoperatively. However, two cases involved the removal of excessively protruding screws and two cases involved bone resection that resolved painful disruption of other joints, such as the ankle. The current patient had proper implant positioning and no other impacted joints, so he was managed without operative intervention. By the final 16-month postoperative follow-up, his symptoms had resolved completely. Although an unusual occurrence with limited data, we recommend nonoperative management for proximal TFJ synostosis caused by tibial nailing if implants are properly positioned and no other joints are affected.

## 1. Introduction

Proximal tibiofibular joint (TFJ) synostosis, as a complication of trauma or surgery, is an exceptionally rare occurrence with few reports available in the literature [1–7]. The literature more commonly reports synostosis occurring in the radioulnar joint [8], the distal TFJ [9, 10], or the tibiofibular diaphyseal area [11]. It also highlights congenital abnormalities and immature skeletal development as a common cause of synostosis [4]. In noncongenital cases, the interosseous bone growth is thought to occur from soft tissue damage, hemorrhage, or subperiosteal dissection [1]. Although rare, noncongenital proximal TFJ synostosis has been reported in two cases of excessive screw protrusion [1, 2] and nine nonsurgical cases [3–7]. Patients with proximal TFJ synostosis may be asymptomatic or they may present with knee pain or painful joint disruptions (e.g., ankle pain) with treatments ranging from nonoperative to screw removal or bone resections [1–7].

## 2. Case Report

**Figure 1:**
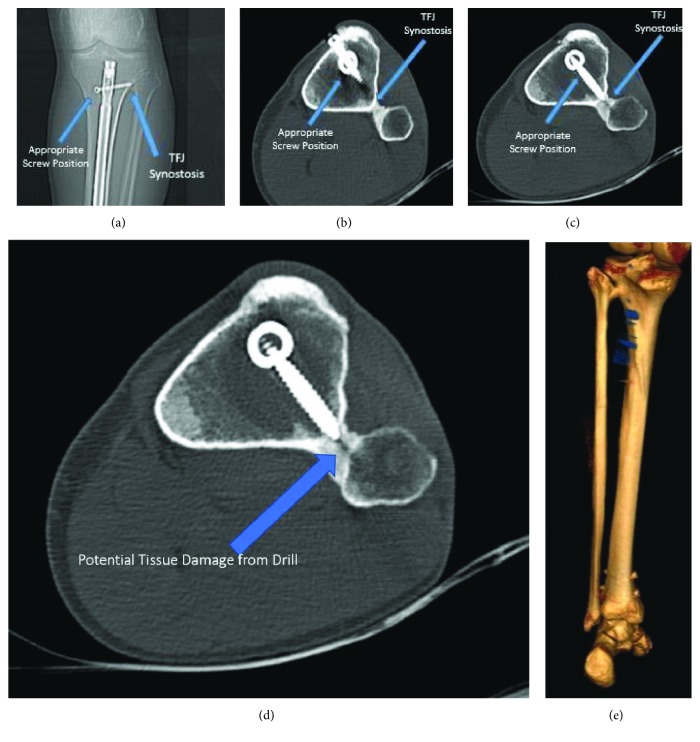
Proximal tibiofibular joint synostosis. (a) AP X-ray of the left proximal tibia show the tibial nail in an acceptable position with visible TFJ synostosis. (b) Axial CT scan showing appropriate position of the implant and TFJ synostosis. (c) Axial CT scan showing appropriate position of interlocking screw without excessive prominence. (d) CT scan demonstrating a potential soft tissue damage from drilling through the TFJ space into the fibula. (e) 3D reconstruction showing TFJ synostosis.

A 28-year-old male semiprofessional basketball player presented to our orthopaedic practice with pain proximal and lateral to the left knee joint. He was treated for a tibial shaft stress fracture with tibial intramedullary nail (IMN) fixation 1 year prior at an outside hospital. He first noticed pain proximal and lateral to the left knee 2 weeks after surgery, and it persisted for the entire year. He had no complaints of pain in other joints and he denied any history of trauma since surgery. X-ray images taken during initial evaluation at our clinic showed no evidence of fractures, appropriate position of hardware, and presence of heterotopic bone at the proximal TFJ (Figure 1(a)). We ordered a CT scan to further evaluate the heterotopic bone and rule out implant problems including screw breakage, loosening, or prominent position. The CT scan demonstrated that the implant was properly positioned with no protruding or loosening screws (Figures 1(a)–1(d)). We posited that the implant likely had little to no impact on the patient's pain. The scan also displayed no acute fracture consistent with the patient's reported level of pain (Figures 1(a)–1(e)). However, it showed that the drill for the proximal locking screw may have penetrated through the tibia and into the fibula (Figure 1(d)). It also revealed proximal tibiofibular synostosis immediately distal to the proximal TFJ joint (Figures 1(a)–1(e)) aligned with the bone reaming along the drill path (Figure 1(d)). Following a literature review and a discussion with our patient, we decided to proceed with nonoperative management. The patient declined a steroid injection. The patient's knee pain resolved without intervention, and he was able to return to playing semiprofessional basketball. At 13 months postoperation, he reported intermittent, mild pain on the medial side of the knee while playing basketball, but this did not limit his participation. At the final follow-up 16 months postoperation, he reported no pain.

## 3. Discussion

Upon presenting with postoperative knee pain near the site of tibial proximal interlocking screws, there was a high suspicion of implant complications. IMN implant issues are very common and the source of many patients' postoperative pain [12, 13], especially with protrusion of interlocking screws beyond 5 mm [14]. While the consensus is not clear in the literature [15, 16], implant removal after tibial IMN may lead to symptomatic relief [12, 13]. The CT scan in the current case failed to show evidence of broken or loose implant that would clearly indicate surgical implant removal. Instead, the CT did show a synostosis aligned with the drill path.

No published case matched our patient's presentation, so we consulted several similar cases to aid in developing a treatment plan for the patient in this case. The most similar case involved a 14-year-old female who developed asymptomatic proximal TFJ synostosis secondary to tibial nailing; the new bone developed around the screw protruding well into the interosseous space. The patient had the screw removed at 48 weeks, and she remained asymptomatic at 5 years. Surgical excision was ruled out while the patient remained asymptomatic [1]. In a second similar case, a 62-year-old female developed asymptomatic proximal TFJ synostosis secondary to an osteotomy when a screw, later removed, penetrated well into the interosseous membrane [2]. While both cases bear similarities to the current case, the CT scan in the current case confirmed appropriate placement of the implant contributing to the decision to continue with nonoperative management.

Nine other noncongenital proximal TFJ synostosis cases were identified in the literature over the last 19 years, although none had a surgical etiology like the current case. Six of the nine identified related cases were asymptomatic and effectively managed nonoperatively [3, 4]. The asymptomatic trend casted doubt on the synostosis as the source of our patient's pain, suggesting that the synostosis acted as a red herring with the pain more likely resulting from the drilled tissue damage. However, in one case, proximal TFJ synostosis was associated with knee and ankle pain presumably by limiting the motion present at each of these joints [5], and in a second case, authors believed proximal TFJ synostosis was associated with referred low back pain (pseudoradicular syndrome) [6]. In both patients, surgical resection separating the tibia from the fibula led to complete symptom resolution [5, 6]. An additional case involved persistent ankle pain, but the patient refused the recommended resection [7]. Nevertheless, in the current case, no such adjacent joint disruption or radicular pain affected the patient, so surgical resection was not further considered.

Knee pain after tibial IMN is a well-known complication with poorly delineated etiology, and it is impossible to attribute the knee pain in the current case to any one factor [17, 18], so the prospect remains that the synostosis or the presence of the implant impacted the pain in the patient. Alternate low probability causes of pain in this case include metal allergies leading to nonspecific deep generalized pain and metal corrosion not seen on imaging [19].

After evaluating factors including imaging of the implant, drilled tissue damage, no painful disruption of adjacent joints, and the asymptomatic nature of the most similar synostosis cases, we surmised that patient's pain was most likely unaffected by the synostosis or the final position of the screw. These considerations along with a patient discussion guided us to the nonoperative approach that coincided with complete symptom resolution 16 months after the operation.

## 4. Conclusion

Proximal TFJ synostosis is an extremely rare finding post IMN fracture fixation. This is the first case where the penetration of the drill into the TFJ without screw prominence likely caused the synostosis and knee pain, the latter of which resolved without intervention. In post-tibial nailing proximal TFJ synostosis, we recommend nonoperative treatment if the screws are not excessively protruding and other joints are not affected pending additional data related to implant removal and this rare form of synostosis.
